# Publisher Correction: Machine learning-guided discovery of ionic polymer electrolytes for lithium metal batteries

**DOI:** 10.1038/s41467-023-39621-z

**Published:** 2023-07-11

**Authors:** Kai Li, Jifeng Wang, Yuanyuan Song, Ying Wang

**Affiliations:** Department of Macromolecular Science, State Key Laboratory of Molecular Engineering of Polymers, Fudan University, Shanghai, 200438 China

**Keywords:** Batteries, Batteries, Polymers, Liquid crystals, Theory and computation

Correction to: *Nature Communications* 10.1038/s41467-023-38493-7, published online 15 May 2023

The original version of this Article contained an error in labels for datapoints in Figure 3e and 3f. A new figure replaces the old figure to amend this error.

Old figure:


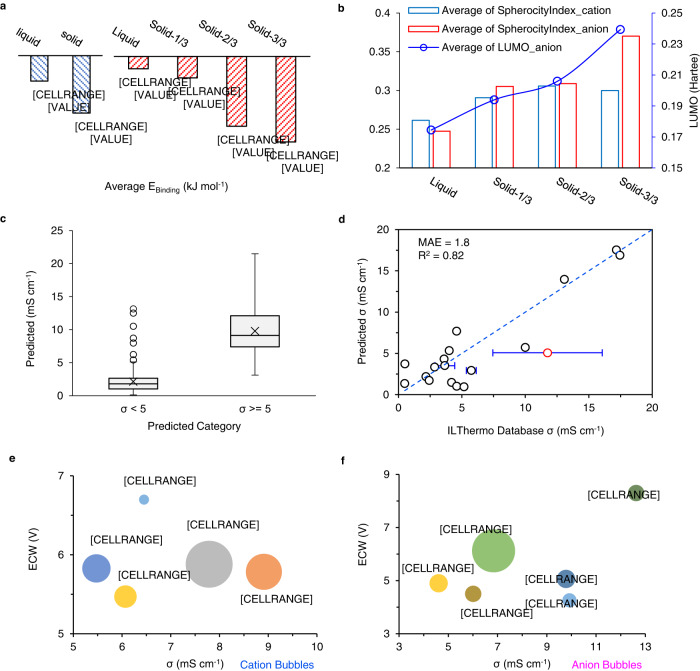


New figure:


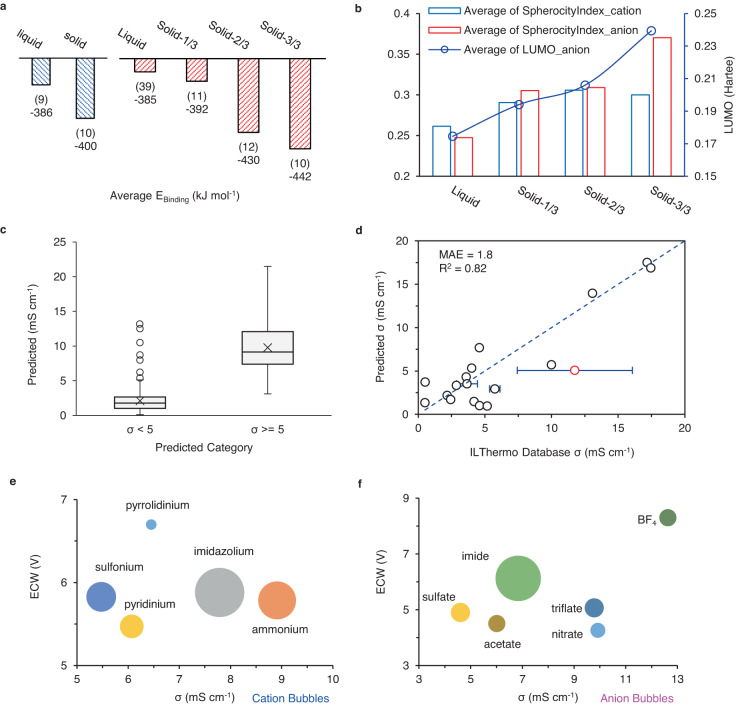


The error has been corrected in the PDF or HTML versions of the Article.

