# Supplementary material for: Machine learning-guided discovery of ionic polymer electrolytes for lithium metal batteries
Source: Nat Commun. 2023 May 15;14:2789. doi: 10.1038/s41467-023-38493-7 (PMC10185508; doi:10.1038/s41467-023-38493-7)
Supplement: Supplementary file 1 — Supplementary Information [file 41467_2023_38493_MOESM1_ESM.pdf]

Supplementary Information

for

Machine learning-guided discovery of ionic polymer electrolytes for lithium metal batteries

Kai Li[1], *Jifeng Wang*[1], *Yuanyuan Song*[1], *Ying Wang*[1]*

1. Department of Macromolecular Science
State Key Laboratory of Molecular Engineering of Polymers
Fudan University
Shanghai, 200438, China
Corresponding E-mail: wying@fudan.edu.cn

Contents

Supplementary Note 1 2
Supplementary Note 2 2
Supplementary Note 3 3
Supplementary Note 4 3
Supplementary Note 5 4
Supplementary Note 6 5
Supplementary Note 7 6
Supplementary Fig. 1. 7
Supplementary Fig. 2 8
Supplementary Fig. 3 9
Supplementary Fig. 4 10
Supplementary Fig. 5 11
Supplementary Fig. 6 12
Supplementary Fig. 7 13
Supplementary Table 1 14
Supplementary Table 2 15
Supplementary Table 3 18
Supplementary Table 4 18
Supplementary Table 5 19
Supplementary Table 6 21
Supplementary Table 7 21

Supplementary Note 1

To investigate the solubility of Li salts, here we introduce the concept of binding energy ($E_{binding}$), which is the lowest energy required to combine the cation(Li^+) and the anions to form the Li-anion pairs. The lower of the $E_{binding}$ means easier for the Li^+ and anions to stay in tightly associated pairs and pack into solid crystals. We observe that the $E_{binding}$ of LiFSI (-512 kJ mol^{-1}) is significantly higher than that of LiTfO (-603 kJ mol^{-1}) and $LiBF_4$ (-602.39 kJ mol^{-1}), this explains the high solubility of LiFSI in ionic liquids compared to the other salts. The $E_{binding}$ is calculated based on Equation 1 in the manuscript. The used theory and basis set is M062X/6-311(+)G(2d,p) with dispersion correction of DFT-D3.

Supplementary Note 2

The Nernst-Einstein equation correlates the conductivity (σ) to the diffusion coeffficents of cation (D^+) and anion (D^-) in the system. The Stokes-Einstein equation displays the relationship among the viscosity (η) ,diffusion coefficients (D) and radii of gyration (r) of the ions or molecules. Thus, we can derive the relationship between the ionic conductivity and the viscosity of the system.

Nernst-Einstein Equation $$\sigma = \frac{z_i^2 F^2}{RT} (D^+ + D^-)$$ Equation (1)

Stoke-Einstein Equation $$D = \frac{k_B T}{6\pi\eta r}$$ Equation (2)

Supplementary Note 3

As indicated in previous literature,[1,2] the uncertainty of the comparison for different cation and anion types is highly related to the limitation of the HOMO/LUMO theory, for example, the C_2mimBF_4 is usually overestimated with a "weak" cation paired with a strong anion. For the cations, the imidazolium type is also not accurate, because the description of the top of the valence band for some of the imidazolium based ILs is not very accurate using the DFT and related approximations, especially for the imidazolium ones with BF_4 anions. However, the overall trend of the ECW values is reliable and showed enough accuracy for screening of potential ILs in this application.

Supplementary Note 4

Here is the list of the 16 factors used for the hierachical clustering of the unsupervised learning. The top 15 factors are obtained from the XGBoosting model for the conductivity classification task. The last factor is ECW_computed calculated based on the HOMO/LUMO theory through Psi4 as described in the manuscript.

1. "MaxPartialCharge_anion",
2. "PMI2_cation",
3. "energy_anion",
4. "RadiusOfGyration_pair",
5. "FpDensityMorgan2_anion",
6. "Eccentricity_pair",
7. "PMI2_pair",

8. "Asphericity_anion",

9. "volume_pair",

10. "Asphericity_pair",

11. "HOMO_cation",

12. "volume_cation",

13. "PMI3_pair",

14. "FpDensityMorgan1_pair",

15. "RadiusOfGyration_anion

16. "'ECW_computed'

Supplementary Note 5

The Graph Convolutional Neural Network (GCN) was built upon the combination of RDkit and PyTorch Geometrics. The RDKit offers the node and edge features of cations and anions. PyTorch takes charge of the train and prediction work. The GCN model as below has two convolutional layers followed with the graph prediction layer. The iteration epoch times is 1000 to ensure stable accuracy values. The code is included in the class object published on GitHub (https://github.com/WangsGroupFDU/IL_machine_learning).

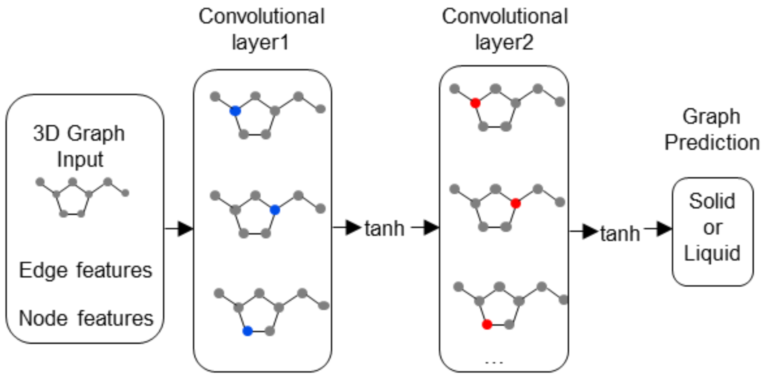

Supplementary Note 6

The R^2 value is widely used to validate the performance of the models based on the predicted values and experimental values. It is very difficult to directly correlate the performance of the model with the R^2 determinant. The R^2 value is highly dependent on many factors, including the sample size, the data sources and the sample uniqueness, thus we have to evaluate case by case.

As far as we know, most of the related literature using machine learning to predict ionic conductivity are based on the ILThemro database, which contains 7234 entries of ionic conductivity values at varying temperatures for only 523 unique ILs, the unique cations and anions are high up to 244 and 109 correspondingly, indicating the database is highly sparse. In addition, we can estimate that there are ~14 (7234/523) records for every unique ionic liquid in the dataset, thus there must be a large number of same ILs appearing both in the training and testing dataset, which will boost the R^2 and lead to the overfitting of the model. Besides, the validation reported in the literature usually relays on the ILThermo database itself. There is seldom validation work using an external database. We didn't find other reported R^2 in the literature, which compares the unique ILs with external database at a single temperature as we report here.

When we do the comparison, 4 ILs contain more than two records as shown in Supplementary Table 6, but the variation between the two records is very high, this uncertainty from the database (experimental values) itself will further increase the fluctuation of our validation shown in Figure 2. Thus, we leave out this invalid value point for 1-butylpyridinium dicyanamide with a huge percentage of difference for the measured values (> 66%). Even though

there are only 18 overlaps between the two datasets, we find that the model did a nice job to predict IL especially with high ionic conductivity, which is one of our targets.

Above all, we conclude that the model is important and the distinctive R^2 value is insightful to the field. This also indicates that we can pay more attention to the commonly existing bias of the database collection and management for future ML investigations.

Supplementary Note 7

The t_{Li}^+ of Bruce-Vincent analysis is defined in Equation (3), where I_0 is the current at the starting point of polarization, I_{ss} is the steady state current at the end of the polarization. R_0 and R_{ss} refer to the initial resistances of the first semi-circle before and after polarization, respectively. The frequency range used is 1Hz – 1MHz. The impedance spectra are attached in Supplementary Table 7.

$$t_{\mathrm{Li}}^{+} = \frac{I_{ss}(\Delta V - I_0 R_0)}{I_0(\Delta V - I_{ss} R_{ss})} \quad \text{Equation (3)}$$

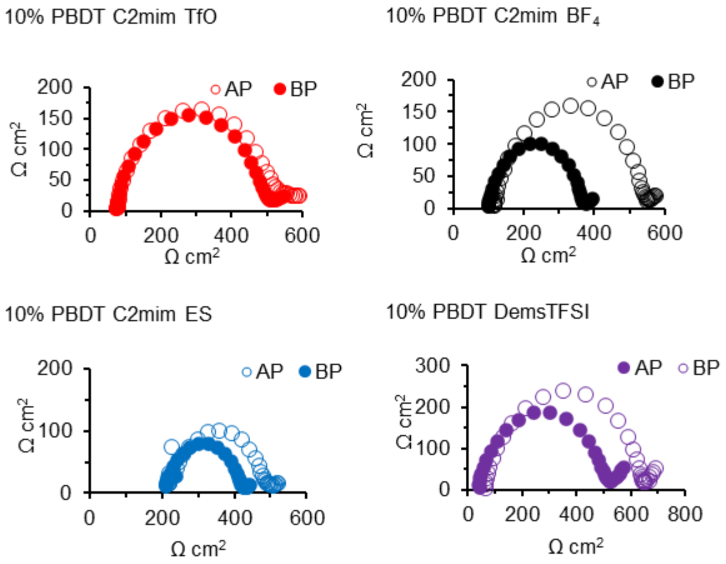

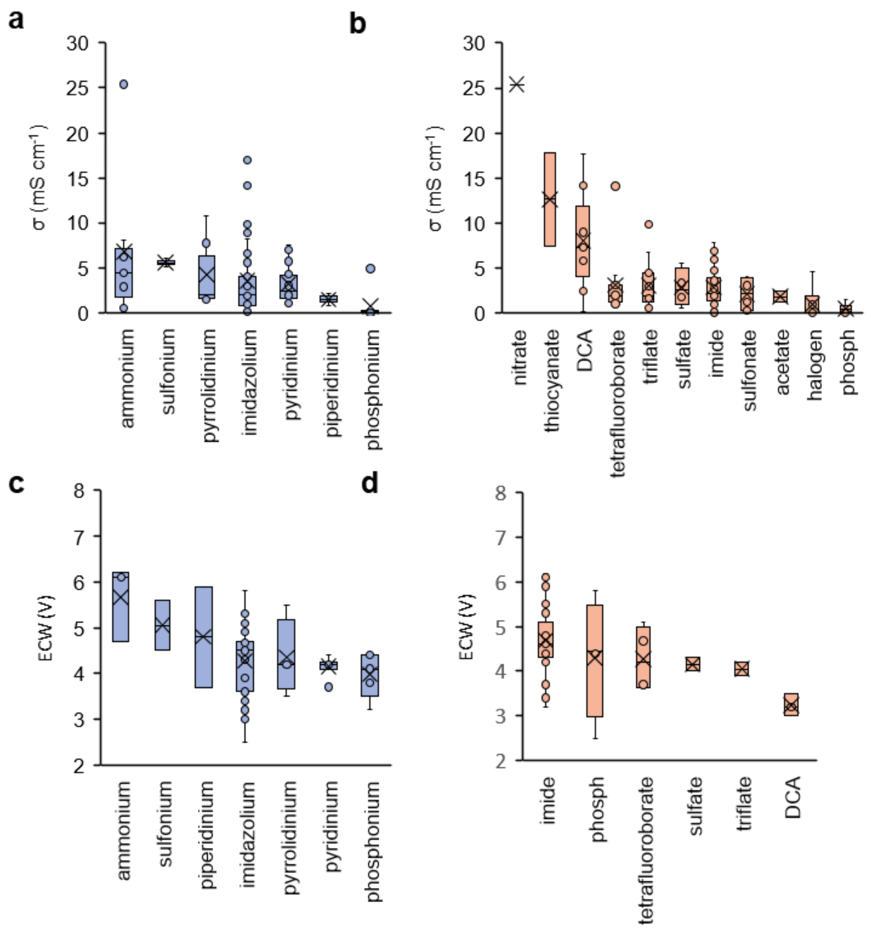

Supplementary Fig. 1.

The conductivity and ECW boxplots for the ILs classified by the cation and anion types. (a, b) The boxplots of the ILs with known conductivity (σ) classified by the cation and anion types, corresponding. In terms of the cations, the average ionic conductivity (σ) follows the order of ammonium > sulfonium > pyrrolidinium > imidazolium > pyridinium > piperidinium > phosphonium. Accordingly, the rule for the anions is nitrate > thiocyanate > DCA > BF4 > triflate > sulfate > imide > sulfonate > acetate > halogen > phosph. (c, d) The boxplots of the ILs with known ECWs classified by the cation and anion types, correspondingly.

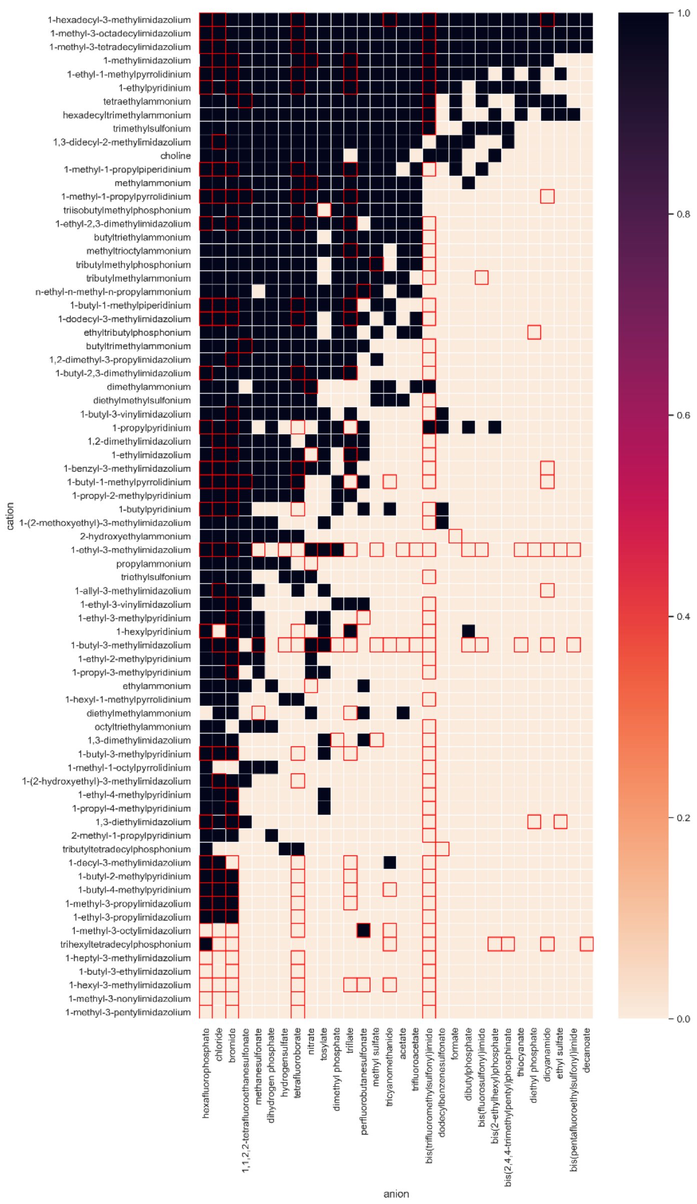

Supplementary Fig. 2

Heatmap for the solid/liquid prediction results as shown in a permutation table between cations and anions. The black squares indicate solid IL pairs at room temperature. The pink squares refer to liquid IL pairs at room temperature. The patch highlighted with red squares are IL pairs with known state in the dataset.

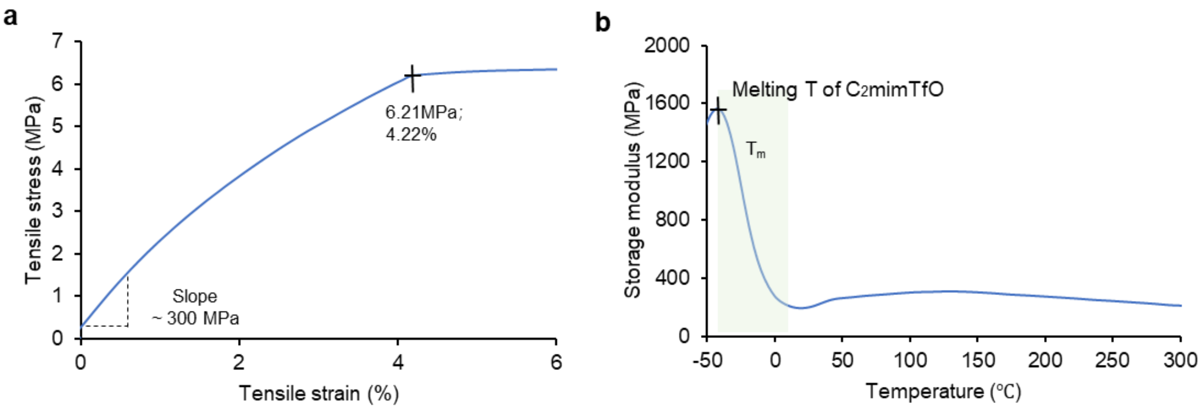

Supplementary Fig. 3

The mechanical properties of IPEs. (a) The stress-strain curve for IPE with 10% PBDT and C2mim TfO. (b) The corresponding DMA curve for IPE with 10% PBDT and C2mim TfO from −50 to 300 °C.

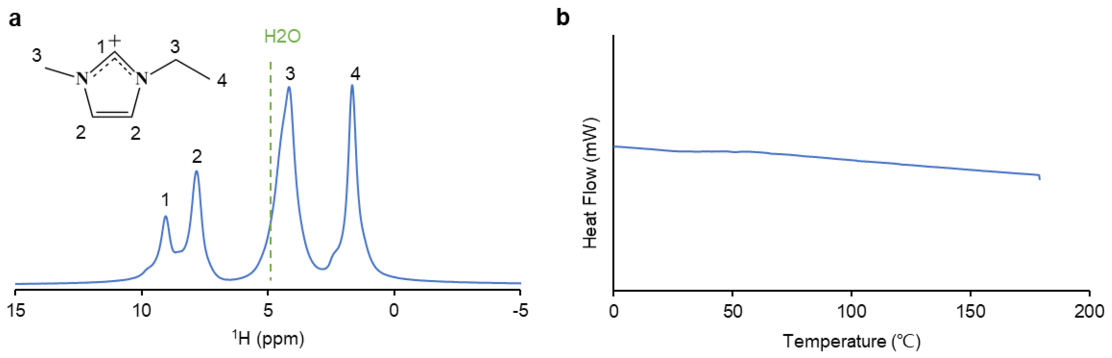

Supplementary Fig. 4

Quantification of H_2O amount in the IPEs using NMR and DSC. (a) ^1H NMR spectra for the membrane. The green dashed line shows the regular position (4.9 ppm) of H_2O peak. H_2O does have a detrimental effect on the performance of Li metal batteries. For this reason, the developed composites membranes of PBDT and ILs formed in the first step were placed in a vacuum oven at 80°C for more than 24h to adequately remove water before assembled in the batteries. Here we mainly need to measure the H_2O in the dried membrane (10% PBDT C2mim TfO). We observe no distinct signal that belongs to H_2O, which usually appears around 4.9 ppm, for the membrane. (b) DSC curve for the membrane. Notably, we observe no apparent heat absorption peaks above 100 °C, which indicates that H_2O molecules were successfully removed after the vacuum drying step. The excellent battery cycling performance in the manuscript also confirms that the effect of H_2O can be neglected in the IPEs. The second ion exchange process was finished in an Ar-filled glove box (< 0.01 ppm H_2O).

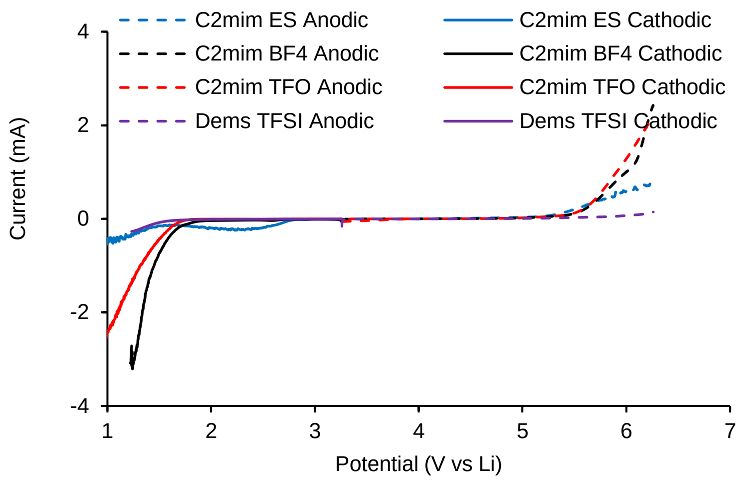

Supplementary Fig. 5

The ECW values for selected pure ILs. The electrochemical window of neat ILs using three electrodes design with Ag|AgCl (3M KCl) as the reference electrode and Pt as both the working and counter electrodes with diameter of 3 mm. The voltage has been converted based on the Li|Li+ potential.

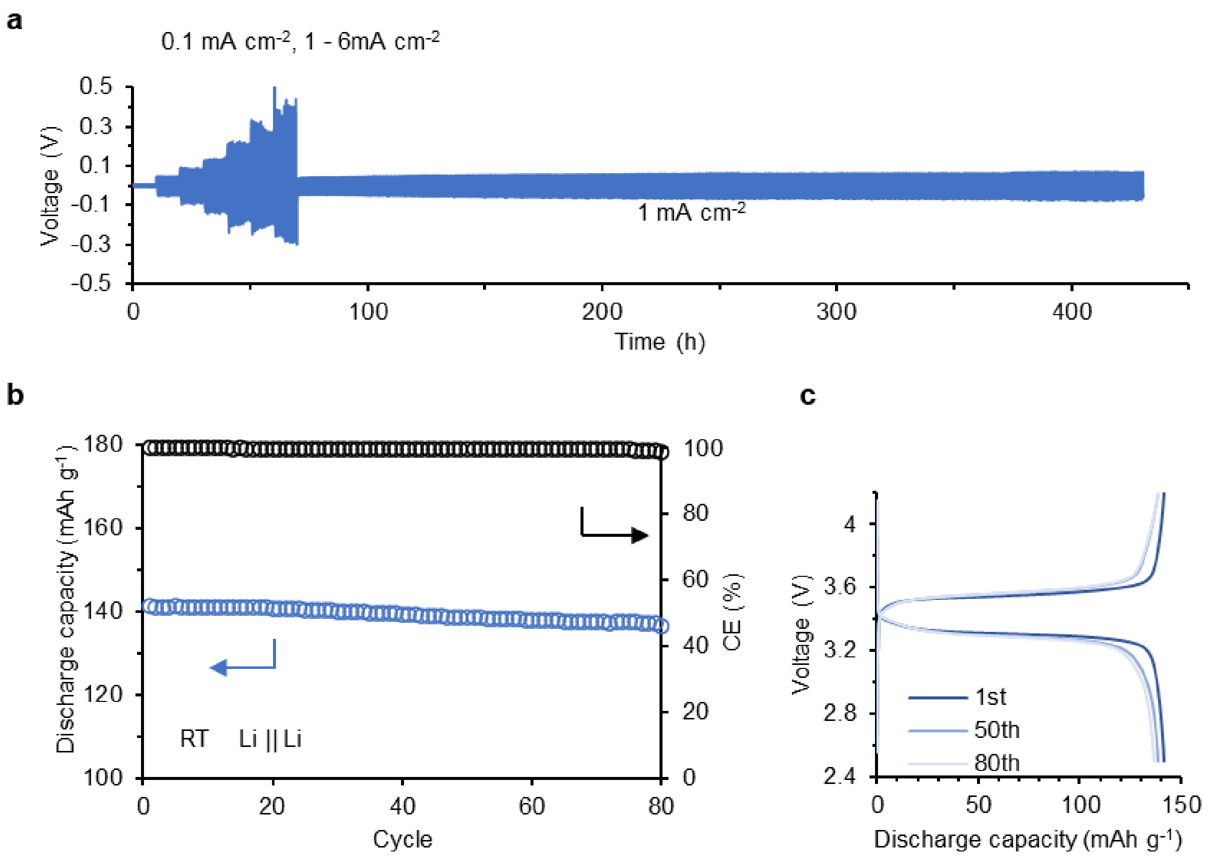

Supplementary Fig. 6

The cycling performance of IPEs based on DemsTFSI. (a) Cell voltage versus time for a symmetric Li|IPEs-DemsTFSI|Li cell at current densities (J) from 0.1 to 6 mA·cm^{-2} with changes in *J* every 10 cycles at room temperature (each cycle lasts 1 h). (b) Cycling performance of Li|IPEs-DemsTFSI|LiFePO4 cell at 0.5C (0.83 mA cm^{-2}) at RT. The blue circles show the specific discharge capacity as a function of the increasing cycle number. The black circles display the CE for each cycle correspondingly. (c) The voltage-capacity profiles for the main cycles in (d).

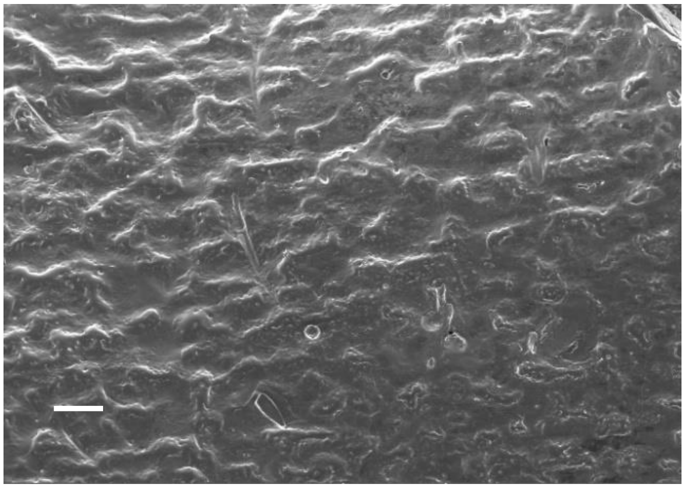

Supplementary Fig. 7

SEM image of the cycled Li metal surface. The scale bar is 100 μm. We observe no Li dendrite formed on the Li metal surface for the Li||Cu cell, which indicates the formation of stable SEI in Li metal surface based on the developed IPEs.

Supplementary Table 1

The calculated binding energies for selected cations and anions pairs (19) labeled with phase at RT from IoLiTec.

cation	anion	$E_{opt}[-]$ (Hartee)	$E_{opt}[+]$ (Hartee)	$E_{opt}[+][-]$ (Hartee)	$E_{binding}$ ($kJmol^{-1}$)	State IoliTec
1-methyl-1-propylpyrrolidinium	chloride	-460.27	-370.14	-830.55	-385	solid
1-butyl-1-methylpiperidinium	chloride	-460.27	-448.75	-909.16	-389	solid
1-ethyl-3-methylimidazolium	chloride	-460.27	-344.48	-804.90	-403	solid
methylammonium	nitrate	-280.35	-96.19	-376.74	-528	solid
1-ethyl-3-methylimidazolium	nitrate	-280.35	-344.48	-624.98	-398	solid
1-ethyl-3-methylimidazolium	tosylate	-894.82	-344.48	-1239.46	-407	solid
1-methyl-1-propylpyrrolidinium	triflate	-961.56	-370.14	-1331.84	-371	solid
1-butyl-1-methylpiperidinium	triflate	-961.56	-448.75	-1410.44	-361	solid
1-methyl-1-propylpyrrolidinium	tetrafluoroborate	-424.56	-370.14	-794.84	-387	solid
1-butyl-1-methylpiperidinium	tetrafluoroborate	-424.56	-448.75	-873.44	-369	solid
1-ethyl-3-methylimidazolium	bis(fluorosulfonyl)imide	-1351.73	-344.48	-1696.35	-358	liquid
1-ethyl-3-methylimidazolium	methanesulfonate	-663.80	-344.48	-1008.44	-415	liquid
1-ethyl-3-methylimidazolium	ethyl sulfate	-778.33	-344.48	-1122.97	-397	liquid
1-ethyl-3-methylimidazolium	thiocyanate	-491.10	-344.48	-835.73	-377	liquid
1-ethyl-3-methylimidazolium	triflate	-961.56	-344.48	-1306.18	-376	liquid
1-ethyl-3-methylimidazolium	tetrafluoroborate	-424.56	-344.48	-769.19	-387	liquid
1-methyl-1-propylpyrrolidinium	dicyanamide	-240.49	-370.14	-610.76	-355	liquid
1-ethyl-3-methylimidazolium	dicyanamide	-240.49	-344.48	-585.11	-369	liquid
1-ethyl-3-methylimidazolium	acetate	-228.51	-344.48	-573.16	-440	liquid

Supplementary Table 2

The calculated binding energies for selected cations and anions pairs (72) labeled with predicted phases at RT.

cation	anion	E_{opt}[-] (Hartee)	E_{opt}[+] (Hartee)	E_{opt}[+][-] (Hartee)	$E_{binding}$ ($kJmol^{-1}$)	State Predict
1-methyl-1-propylpyrrolidinium	bis(fluorosulfonyl)imide	-1351.73	-370.14	-1722.00	-347	Liquid
1-butyl-1-methylpiperidinium	bis(fluorosulfonyl)imide	-1351.73	-448.75	-1800.61	-342	Liquid
diethylmethylsulfonium	bis(fluorosulfonyl)imide	-1351.73	-556.94	-1908.80	-353	Liquid
1-ethyl-3-methylpyridinium	bis(fluorosulfonyl)imide	-1351.73	-366.54	-1718.39	-337	Liquid
ethyltributylphosphonium	bis(fluorosulfonyl)imide	-1351.73	-893.80	-2245.66	-330	Liquid
ethyltributylphosphonium	nitrate	-280.35	-893.80	-1174.30	-382	Liquid
1-ethyl-3-methylimidazolium	dihydrogen phosphate	-643.63	-344.48	-988.28	-440	Liquid
ethyltributylphosphonium	dihydrogen phosphate	-643.63	-893.80	-1537.60	-435	Liquid
ethyltributylphosphonium	methanesulfonate	-663.80	-893.80	-1557.76	-400	Liquid
1-ethyl-3-methylpyridinium	tosylate	-894.82	-366.54	-1261.51	-391	Liquid
ethyltributylphosphonium	tosylate	-894.82	-893.80	-1788.78	-389	Liquid
methylammonium	ethyl sulfate	-778.33	-96.19	-874.71	-496	Liquid
1-methyl-1-propylpyrrolidinium	ethyl sulfate	-778.33	-370.14	-1148.62	-385	Liquid
1-butyl-1-methylpiperidinium	ethyl sulfate	-778.33	-448.75	-1227.23	-389	Liquid
diethylmethylsulfonium	ethyl sulfate	-778.33	-556.94	-1335.42	-405	Liquid
1-ethyl-3-methylpyridinium	ethyl sulfate	-778.33	-366.54	-1145.02	-393	Liquid
ethyltributylphosphonium	ethyl sulfate	-778.33	-893.80	-1672.28	-384	Liquid

methylammonium	thiocyanate	-491.10	-96.19	-587.47	-466	Liquid
1-methyl-1-propylpyrrolidinium	thiocyanate	-491.10	-370.14	-861.38	-376	Liquid
1-butyl-1-methylpiperidinium	thiocyanate	-491.10	-448.75	-939.99	-370	Liquid
diethylmethylsulfonium	thiocyanate	-491.10	-556.94	-1048.18	-383	Liquid
1-ethyl-3-methylpyridinium	thiocyanate	-491.10	-366.54	-857.78	-365	Liquid
ethyltributylphosphonium	thiocyanate	-491.10	-893.80	-1385.04	-354	Liquid
ethyltributylphosphonium	triflate	-961.56	-893.80	-1855.50	-358	Liquid
1-ethyl-3-methylpyridinium	tetrafluoroborate	-424.56	-366.54	-791.24	-376	Liquid
1-ethyl-3-methylimidazolium	tricyanomethanide	-316.69	-344.48	-661.31	-356	Liquid
diethylmethylsulfonium	tricyanomethanide	-316.69	-556.94	-873.75	-331	Liquid
1-ethyl-3-methylpyridinium	tricyanomethanide	-316.69	-366.54	-683.36	-343	Liquid
ethyltributylphosphonium	tricyanomethanide	-316.69	-893.80	-1210.62	-327	Liquid
methylammonium	dicyanamide	-240.49	-96.19	-336.85	-452	Liquid
1-butyl-1-methylpiperidinium	dicyanamide	-240.49	-448.75	-689.37	-355	Liquid
diethylmethylsulfonium	dicyanamide	-240.49	-556.94	-797.56	-356	Liquid
1-ethyl-3-methylpyridinium	dicyanamide	-240.49	-366.54	-607.17	-364	Liquid
ethyltributylphosphonium	dicyanamide	-240.49	-893.80	-1134.43	-349	Liquid
1-methyl-1-propylpyrrolidinium	acetate	-228.51	-370.14	-598.81	-438	Liquid
1-butyl-1-methylpiperidinium	acetate	-228.51	-448.75	-677.41	-420	Liquid
diethylmethylsulfonium	acetate	-228.51	-556.94	-785.61	-438	Liquid
1-ethyl-3-methylpyridinium	acetate	-228.51	-366.54	-595.20	-412	Liquid
ethyltributylphosphonium	acetate	-228.51	-893.80	-1122.47	-416	Liquid
ethyltributylphosphonium	chloride	-460.27	-893.80	-1354.22	-387	Solid-1

methylammonium	bis(fluorosulfonyl)imide	-1351.73	-96.19	-1448.08	-430	Solid-1
diethylmethylsulfonium	dihydrogen phosphate	-643.63	-556.94	-1200.73	-448	Solid-1
diethylmethylsulfonium	methanesulfonate	-663.80	-556.94	-1220.90	-422	Solid-1
1-ethyl-3-methylpyridinium	methanesulfonate	-663.80	-366.54	-1030.49	-406	Solid-1
1-methyl-1-propylpyrrolidinium	tosylate	-894.82	-370.14	-1265.11	-380	Solid-1
1-butyl-1-methylpiperidinium	tosylate	-894.82	-448.75	-1343.71	-356	Solid-1
diethylmethylsulfonium	tosylate	-894.82	-556.94	-1451.92	-409	Solid-1
diethylmethylsulfonium	triflate	-961.56	-556.94	-1518.63	-376	Solid-1
1-ethyl-3-methylpyridinium	triflate	-961.56	-366.54	-1328.23	-368	Solid-1
1-butyl-1-methylpiperidinium	tricyanomethanide	-316.69	-448.75	-765.56	-329	Solid-1
methylammonium	chloride	-460.27	-96.19	-556.65	-525	Solid-2
1-methyl-1-propylpyrrolidinium	nitrate	-280.35	-370.14	-650.64	-385	Solid-2
1-butyl-1-methylpiperidinium	nitrate	-280.35	-448.75	-729.24	-384	Solid-2
diethylmethylsulfonium	nitrate	-280.35	-556.94	-837.44	-404	Solid-2
1-ethyl-3-methylpyridinium	nitrate	-280.35	-366.54	-647.04	-390	Solid-2
1-ethyl-3-methylpyridinium	dihydrogen phosphate	-643.63	-366.54	-1010.33	-425	Solid-2
1-butyl-1-methylpiperidinium	Methane sulfonate	-663.80	-448.75	-1112.70	-398	Solid-2
methylammonium	tosylate	-894.82	-96.19	-991.20	-501	Solid-2
diethylmethylsulfonium	tetrafluoroborate	-424.56	-556.94	-981.64	-389	Solid-2
methylammonium	tricyanomethanide	-316.69	-96.19	-413.03	-404	Solid-2
1-methyl-1-propylpyrrolidinium	tricyanomethanide	-316.69	-370.14	-686.95	-332	Solid-2
methylammonium	acetate	-228.51	-96.19	-324.93	-623	Solid-2
diethylmethylsulfonium	chloride	-460.27	-556.94	-1017.36	-418	Solid-3
1-ethyl-3-	chloride	-460.27	-366.54	-826.95	-386	Solid-3

methylpyridinium						
methylammonium	dihydrogen phosphate	-643.63	-96.19	-740.03	-575	Solid-3
1-methyl-1-propylpyrrolidinium	dihydrogen phosphate	-643.63	-370.14	-1013.93	-416	Solid-3
1-butyl-1-methylpiperidinium	dihydrogen phosphate	-643.63	-448.75	-1092.54	-417	Solid-3
methylammonium	Methane sulfonate	-663.80	-96.19	-760.18	-517	Solid-3
1-methyl-1-propylpyrrolidinium	Methane sulfonate	-663.80	-370.14	-1034.09	-393	Solid-3
methylammonium	triflate	-961.56	-96.19	-1057.92	-463	Solid-3
methylammonium	tetrafluoroborate	-424.56	-96.19	-520.92	-471	Solid-3
ethyltributylphosphonium	tetrafluoroborate	-424.56	-893.80	-1318.50	-369	Solid-3

Supplementary Table 3

ANOVA results for the hypothesis testing.

Source of Variation	*SS*	*df*	*MS*	*F*	*P-value*	*F crit*
Between Groups	38521.58	3	12840.53	4.77142	0.004459	2.739502
Within Groups	182997.1	68	2691.134			
Total	221518.7	71				

Supplementary Table 4

T-test with unequal variance results for the hypothesis testing.

t-test	*p-val(one_tail)*	*t_Stat*	*t_Critical*	*df*
Liquid vs. Solid-1/3	0.299	0.53	1.667	48
**Liquid vs. Solid-2/3**	**0.0057**	**2.63**	**1.676**	**49**
**Liquid vs. Solid-3/3**	**0.00047**	**3.53**	**1.678**	**47**
**Liquid vs. Solid-all**	**0.002559**	**2.89**	**1.667**	**70**

Supplementary Table 5

The final recommendation list with 49 ILs for IPEs.

Pair	ECW (V)	σ (mS cm^{-1})	Cation	Anion
1-ethyl-3-methylpyridinium bis(trifluoromethylsulfonyl)imide	5.0	6.2	pyridinium	imide
1-ethyl-3-methylpyridinium bis(fluorosulfonyl)imide	5.4	6.2	pyridinium	imide
1,3-diethylimidazolium bis(trifluoromethylsulfonyl)imide	6.1	6.9	imidazolium	imide
1,3-diethylimidazolium bis(fluorosulfonyl)imide	6.6	6.8	imidazolium	imide
1,3-dimethylimidazolium bis(trifluoromethylsulfonyl)imide	6.0	7.8	imidazolium	imide
1,3-dimethylimidazolium ethyl sulfate	4.9	4.1	imidazolium	sulfate
1,3-dimethylimidazolium trifluoroacetate	4.5	6.5	imidazolium	acetate
1,3-dimethylimidazolium tetrafluoroborate	8.6	12.9	imidazolium	tetrafluo roborate
1,3-dimethylimidazolium bis(fluorosulfonyl)imide	6.5	7.9	imidazolium	imide
1-(2-hydroxyethyl)-3-methylimidazolium nitrate	4.3	10.1	imidazolium	nitrate
1-allyl-3-methylimidazolium bis(fluorosulfonyl)imide	6.6	7.3	imidazolium	imide
1-ethyl-3-methylimidazolium bis(trifluoromethylsulfonyl)imide	6.1	6.6	imidazolium	imide
1-ethyl-3-methylimidazolium triflate	5.5	9.8	imidazolium	triflate
1-ethyl-3-methylimidazolium ethyl sulfate	4.9	5.6	imidazolium	sulfate
1-ethyl-3-methylimidazolium trifluoroacetate	4.5	6.1	imidazolium	acetate
1-ethyl-3-methylimidazolium tetrafluoroborate	8.6	14.1	imidazolium	tetrafluo roborate
1-ethyl-3-methylimidazolium bis(fluorosulfonyl)imide	6.5	8.3	imidazolium	imide
1-methyl-1-propylpyrrolidinium bis(fluorosulfonyl)imide	6.7	6.5	pyrrolidiniu m	imide
1-methyl-3-propylimidazolium bis(trifluoromethylsulfonyl)imide	6.1	5.5	imidazolium	imide
1-methyl-3-propylimidazolium triflate	5.6	6.1	imidazolium	triflate
1-methyl-3-propylimidazolium bis(fluorosulfonyl)imide	6.5	6.5	imidazolium	imide
1-propyl-3-methylpyridinium bis(trifluoromethylsulfonyl)imide	5.0	6.2	pyridinium	imide
1-propyl-3-methylpyridinium	5.5	5.6	pyridinium	imide

bis(fluorosulfonyl)imide				
2-hydroxyethylammonium bis(fluorosulfonyl)imide	5.6	8.9	ammonium	imide
butyltrimethylammonium bis(fluorosulfonyl)imide	6.6	5.6	ammonium	imide
diethylmethylammonium triflate	5.5	6.7	ammonium	triflate
diethylmethylammonium ethyl sulfate	4.8	3.7	ammonium	sulfate
diethylmethylammonium trifluoroacetate	4.4	6.3	ammonium	acetate
diethylmethylammonium tetrafluoroborate	8.5	8.4	ammonium	tetrafluo roborate
diethylmethylammonium bis(fluorosulfonyl)imide	6.4	6.6	ammonium	imide
diethylmethylsulfonium bis(trifluoromethylsulfonyl)imide	6.0	6.0	sulfonium	imide
diethylmethylsulfonium ethyl sulfate	4.8	5.8	sulfonium	sulfate
diethylmethylsulfonium bis(fluorosulfonyl)imide	6.4	8.4	sulfonium	imide
ethylammonium triflate	4.4	16.7	ammonium	triflate
ethylammonium bis(fluorosulfonyl)imide	5.3	12.5	ammonium	imide
triethylsulfonium bis(trifluoromethylsulfonyl)imide	6.2	5.1	sulfonium	imide
triethylsulfonium ethyl sulfate	5.1	3.8	sulfonium	sulfate
triethylsulfonium bis(pentafluoroethylsulfonyl)imide	6.7	3.1	sulfonium	imide
triethylsulfonium trifluoroacetate	4.7	5.2	sulfonium	acetate
triethylsulfonium bis(fluorosulfonyl)imide	6.7	6.4	sulfonium	imide
1,2-dimethylimidazolium bis(fluorosulfonyl)imide	6.3	5.2	imidazolium	imide
1-methyl-3-pentylimidazolium nitrate	4.4	4.0	imidazolium	nitrate
1-ethylimidazolium nitrate	4.1	15.6	imidazolium	nitrate
1-ethylimidazolium bis(fluorosulfonyl)imide	6.3	7.5	imidazolium	imide
1-propyl-2-methylpyridinium bis(fluorosulfonyl)imide	6.5	6.1	pyridinium	imide
n-ethyl-n-methyl-n-propylammonium bis(fluorosulfonyl)imide	6.4	6.9	ammonium	imide
propylammonium triflate	4.4	9.5	ammonium	triflate
propylammonium tetrafluoroborate	7.5	15.1	ammonium	tetrafluo roborate
propylammonium bis(fluorosulfonyl)imide	5.4	8.8	ammonium	imide

Supplementary Table 6

The conductivity records stored in ILThermo for the 18 overlapped ILs.

Number	Label	Conductivity (mS cm^{-1}) 25°C		
		Record 1	Record 2	Record 3
1	1-butyl-3-methylimidazolium trifluoroacetate	3.1	3.32	4.55
2	1-butylpyridinium dicyanamide	8.7	14.8	
3	1-ethyl-3-methylimidazolium methyl sulfate	5.47	6.02	
4	propylammonium acetate	0.43	0.6	
5	1,2-dimethyl-3-propylimidazolium thiocyanate	4.59		
6	1,3-dimethylimidazolium acetate	2.86		
7	1-butyl-2,3-dimethylimidazolium thiocyanate	2.17		
8	1-butyl-3-ethylimidazolium bromide	0.502		
9	1-butyl-3-methylimidazolium dihydrogen phosphate	4.19		
10	1-ethyl-3-methylimidazolium trifluoroacetate	10		
11	1-ethylpyridinium dicyanamide	17.18		
12	1-hexyl-3-methylimidazolium dicyanamide	5.17		
13	1-hexylpyridinium dicyanamide	4.6		
14	1-methyl-3-pentylimidazolium dihydrogen phosphate	2.43		
15	1-methyl-3-propylimidazolium dicyanamide	17.46		
16	1-propylpyridinium dicyanamide	13.08		
17	1-propylpyridinium tetrafluoroborate	4.01		
18	propylammonium formate	3.6		

Supplementary Table 7

The t_{Li}^+ of Bruce-Vincent analysis of the IPEs developed from different ILs.

IPEs	I_{ss} (μA)	I_0(μA)	R_{ss}(Ω)	R_0(Ω)	Polarization (mV)	t_{Li}^+
10% PBDT C2mim TfO	9.97	18.8	470	441	10	0.5
10% PBDT C2mim BF4	9.54	26.2	450	270	10	0.4
10% PBDT C2mim ES	9.54	23.0	295	223	10	0.4
10% PBDT Dems TFSI	25.8	50.9	478	580	10	0.5

1 Ong, S. P., Andreussi, O., Wu, Y., Marzari, N. & Ceder, G. Electrochemical Windows of Room-Temperature Ionic Liquids from Molecular Dynamics and Density Functional Theory Calculations. *Chem Mater* **23**, 2979-2986, doi:10.1021/cm200679y (2011).

2 Kuusik, I., Kook, M., Pärna, R. & Kisand, V. Ionic Liquid Vapors in Vacuum: Possibility to Derive Anodic Stabilities from DFT and UPS. *ACS omega* **6**, 5255-5265, doi:10.1021/acsomega.0c05369 (2021).
